# Comparison of Treatment Outcomes of Surgical Repair in Inguinal Hernia with Classic versus Preperitoneal Methods on Reduction of Postoperative Complications

**DOI:** 10.1155/2017/3785302

**Published:** 2017-01-23

**Authors:** Hormoz Mahmoudvand, Shahab Forutani, Sedigheh Nadri

**Affiliations:** ^1^Department of Surgery, Lorestan University of Medical Sciences, Khorramabad, Iran; ^2^Medical Student Research Committee, Lorestan University of Medical Sciences, Khorramabad, Iran; ^3^Department of Anesthesiology, Lorestan University of Medical Sciences, Khorramabad, Iran

## Abstract

*Background*. This study aims to evaluate and compare the results of inguinal herniorrhaphy with mesh in classic and preperitoneal method.* Methods*. Our study community includes 150 candidate patients for inguinal herniorrhaphy with mesh. Totally, 150 candidate patients for inguinal herniorrhaphy were randomly divided into two groups: (1) classic group in which the floor of the canal was repaired and the mesh was located on the floor of the canal and (2) preperitoneal group in which the mesh was installed under the canal and then the floor was repaired.* Results*. The frequency of recurrence was 10 (13.3%) and 2 (2.66%) in the classic and preperitoneal group, respectively. The frequency of postsurgical pain was 21 (28%) in the classic group and 9 (12%) in the preperitoneal group. The postsurgical hematoma was observed in 7 (9.3%) and 9 (12%) in the classic and preperitoneal group, respectively. Also, the frequency of postsurgical seroma was 8 (10.7%) and 1 (1.3%) in the patients treated with the classic and preperitoneal method, respectively.* Conclusion*. The findings of the present study demonstrated that the preperitoneal method is a more suitable method for inguinal herniorrhaphy than the classic one because of fewer complications, according to the findings of this study.

## 1. Introduction

Hernia generally means weakness or defect of the body wall muscle fibers that provide a space for protrusion of internal organs [1]. According to the previous studies, prevalence of the inguinal hernia is nearly 5% worldwide [2]. In the United States, 700,000 herniorrhaphy procedures are performed annually, which shows the high prevalence of the disease [3]. Inguinal hernia is divided into two categories, direct and indirect, which include 24 and 50 percent of all types of hernia, respectively [4]. Moreover, ventral hernia and femoral hernia covered approximately 10 and 3% of cases, respectively. A small percentage of hernia relates to uncommon hernias [5]. If hernia can be pushed back by the maneuvers, it is called reducible. Otherwise, it is called irreducible.

If there is no blood flow in the viscera sticking in the hernia, hernia is called congested or strangulated [6]. The causing and predisposing factors of this condition are not known clearly but the factors that increase the pressure in the abdominal wall are mentioned. For example, chronic cough, chronic obstructive pulmonary disease, chronic constipation, benign prostatic hyperplasia, family history of hernia, collagen diseases, previous right lower quadrant incision, smoking, physical activity, and bearing the burden may be named [7, 8].

Surgical treatment is the choice treatment of this disorder. Today, there are various methods of surgery and the chief goal of treatment is to heal patients and reduce the recurrence of disease. The prolene meshes have reduced the recurrence greatly in the last 20 years [9, 10]. There are two main methods for surgery: open surgery and laparoscopy. There are various methods to repair the herniation site, two of which are more applicable: classical and preperitoneal methods. The classical method is an easier method than other methods of repair performed by most surgeons and it is the gold standard of herniorrhaphy [11–13]. In this method, the mesh is located on the floor of the inguinal canal, below which the thin transverse abdominis fascia is placed. So, it causes a relapse-prone area. However, the recurrence rates reduce in the preperitoneal method because the mesh is laid under the fascia and on the peritoneum [14, 15].

This study aims to evaluate and compare the results of inguinal herniorrhaphy with mesh in classic and preperitoneal method because of the high incidence of complications after inguinal herniorrhaphy and the variety of reconstructive procedures, in general hospitals, Lorestan province, western Iran.

## 2. Materials and Methods

### 2.1. Ethical Statement

This study was approved by the Ethics Committee of Lorestan University of Medical Sciences (permit number 90/236). In addition, written informed consent was obtained from all the participants before surgery.

### 2.2. Patients

This randomized clinical trial was conducted on 150 candidate patients for inguinal herniorrhaphy with mesh.

Subjects enrolled with personal satisfaction. Both methods were explained to them. Given that these two are pretty standard procedures, no specific complication will occur. However, the surgical team accepted any responsibilities if there were any problems. It should be noted that the patients had the possibility of withdrawal from the study in the disinclination to continue in participating in this study.

#### 2.2.1. Surgical Procedure

Patients were randomly assigned to two treatment groups. Duration for surgeries was approximately between 30 and 45 minutes. The patients underwent a surgical repair in inguinal hernia with classic versus preperitoneal methods under spinal anesthesia. In both groups, the surgeon incised the skin and subcutaneous tissue of the lower part of the abdomen and then the fascia of Scarpa and the roof of the inguinal canal. The first group was assigned to the classic method; after reinforcement of the posterior wall of the inguinal canal, the Mersilene mesh (7.5 × 10 cm) was placed and fixed using Round nylon stitch 3/0 to the edges of the defect or weakness in the posterior wall. The second group was assigned to the preperitoneal method; briefly, after acquiring the posterior wall of the inguinal canal, the Mersilene mesh (7.5 × 10 cm) was placed and fixed using Round nylon stitch 3/0 under the posterior wall and then was rehabilitated based on modified Bassini repair method. All patients were followed up for 6–12 months after surgery.

Inclusion criteria include having direct hernia with defects in the posterior wall, being a candidate for classic herniorrhaphy, being a candidate for preperitoneal herniorrhaphy, and satisfaction to enter the study. Exclusion criteria include diabetes, bleeding disorders, and aspirin and corticosteroid consumption. Both groups were compared after surgery in terms of recurrence, pain, seroma, and hematoma in 3- to 12-month periods.

### 2.3. Statistical Analysis

The data were analyzed by IBM SPSS Statistics 23. The differences in the variables were determined by the Chi-Squared test and Fisher's exact test between classic and preperitoneal methods. Overall, *p* < 0.05 was proposed to represent statistical significance after correction.

## 3. Results

From 150 patients, 75 were assigned to the classic method and 75 were assigned to the preperitoneal method. In the classic group, 64% were male and 36% were female. In the preperitoneal group, 54.7% were male and 45.3 were female; the difference was not significant according to Chi-Squared test (*p* = 0.245) (Table 1).

The rate of recurrence was 10 (13.3%) in the classic group and 2 (2.66%) in the preperitoneal group. This difference was significant according to Chi-Squared test (*p* = 0.016) (Table 2).

The frequency of postoperative pain was 21 (28%) in the classic group and 9 (12%) in the preperitoneal group. This difference was significant according to Chi-Squared test (*p* = 0.014) (Table 3).

The frequency of postsurgical hematoma was 7 (9.3%) in the classic group and 9 (12%) in the preperitoneal group. This difference was not significant according to Chi-Squared test (*p* = 0.597) (Table 4).

The rate of postsurgical seroma was 8 (10.7%) in the patients treated with the classic method. This value was 1 (1.3%) with the preperitoneal method; hence, this difference was significant according to Fisher's exact test (*p* = 0.034) (Table 5).

## 4. Discussion

Inguinal hernia repair (also referred to as herniorrhaphy or hernioplasty) is one of the most frequently performed surgical actions worldwide. Nowadays, the majority of surgeons choose to carry out a tension-free mesh repair. Various aspects of postoperative complications of herniorrhaphy were discussed in several studies. In the study conducted by Khoshnevis and Falah on the results and complications of Bassini methods and Lichtenstein and Bassini methods with mesh in Shohadaye Tajrish Hospital in Tehran (Iran), it was concluded that both Bassini and Liechtenstein methods have similar complications and recurrence. However, the Bassini approach may be more appropriate for inguinal hernia repair in less developed countries because it is less expensive [16]. Also, the recurrence rate had no significant difference in the classic and preperitoneal methods according to the study of Muldoon and colleagues in 2004. This amount was reported to be 4.3% and less than 1%, respectively [15].

Other studies described the effect of postoperative pain. In Moghaddam et al.'s study, the pain of operation site was lower in the preperitoneal method than in the classical method. However, the classic method is a simpler procedure but pain is higher in this type of operation, which may be due to direct contact of the spermatic cord with the mesh. In contrast, the pain of operation site was lower in the preperitoneal method because the mesh was inserted with fewer sutures under the transversalis fascia [17]. In another study, Khorshidi et al. investigated the effect of the use of morphine and bupivacaine on the length of hospitalization. The results demonstrated that ilioinguinal and iliohypogastric nerve block by bupivacaine can reduce the need for morphine and hospitalization after surgery. Therefore, this method can be used to control postsurgical pain [18].

In this study, we discussed the open classic and preperitoneal methods. Mesh is used in both of these methods. In a study, the mean scores of quality of life including physical and mental health were almost similar in all methods with mesh but they have a significant difference in comparison with the tissue repair method [19]. Therefore, we claim that the method of repair with mesh is a better method than the tissue method.

The rate of recurrence, postoperative pain, and hematoma was significantly lower in the preperitoneal group compared with the classic one in this study. Perhaps this was due to the insertion of mesh under the transverse fascia and on the peritoneum in the preperitoneal method. Surely, the preperitoneal method makes less weak areas in the wall of the repaired site than the classic one in which mesh is placed on the fascia. Also, the pain is higher in the classic method, which may be due to direct contact of the mesh with the spermatic cord.

Finally, it seems that the preperitoneal method is a more suitable method for inguinal herniorrhaphy than the classic one because of fewer complications, according to the findings of this study. It should be noted that the determination of the type of operation needs a lot of benchmarks, and medical staffs should perform the most appropriate procedure according to all aspects to treat the patients.

## Figures and Tables

**Table 1:** Comparison of sexual frequency distribution of the study groups.

Study group	Sex			*p* value
	Male	Female	Total	
	Number (%)	Number (%)	Number (%)	
Classic	64 (48)	27 (36)	75 (100)	0.245
Preperitoneal	41 (54.7)	34 (45.3)	75 (100)	

**Table 2:** Frequency distribution of recurrence in the study groups.

Study group	Recurrence			*p* value
	Yes	No	Total	
	Number (%)	Number (%)	Number (%)	
Classic	10 (13.3)	65 (86.7)	75 (100)	0.016
Preperitoneal	2 (2.66)	73 (97.33)	75 (100)	

**Table 3:** Comparison of frequency distribution of pain in the study groups.

Study group	Pain after surgery			*p* value
	Yes	No	Total	
	Number (%)	Number (%)	Number (%)	
Classic	21 (28)	54 (72)	75 (100)	0.014
Preperitoneal	9 (12)	66 (88)	75 (100)	

**Table 4:** Comparison of the frequency of postoperative hematoma in the study groups.

Study group	Hematoma			*p* value
	Yes	No	Total	
	Number (%)	Number (%)	Number (%)	
Classic	7 (9.3)	68 (90.7)	75 (100)	0.597
Preperitoneal	9 (12)	66 (88)	75 (100)	

**Table 5:** Comparison of the frequency of postoperative seroma in the study groups.

Study group	Seroma			*p* value
	Yes	No	Total	
	Number (%)	Number (%)	Number (%)	
Classic	8 (10.7)	67 (89.3)	75 (100)	0.034
Preperitoneal	1 (1.3)	74 (98.7)	75 (100)	

## References

[1] Kingsnorth A., LeBlanc K. (2003). Hernias: inguinal and incisional. *The Lancet*.

[2] Dattola P., Alberti A., Dattola A., Giannetto G., Basile G., Basile M. (2002). Inguino-crural hernias: preoperative diagnosis and post-operative follow-up by high-resolution ultrasonography. A personal experience. *Annali Italiani di Chirurgia*.

[3] Rutkow I. M., Arreyui M. E., Nagan R. F. (1994). Open versus Laparoscopic groin herniorhaphy: economic realities. *Inguinal Hernia: Advances or Contraversies?*.

[4] Njhus L. M., Coclon R. E., Judge C., Rhoads J. E. (1989). *Hernia*.

[5] Eubanks W. S., Hern I. U., Townsend C. M., Beauchamp R. D., Evers B. M., Mattox K. L. (2001). *Sabiston Textbook of Surgery the Biological Basis of Modem Surgical Practice*.

[6] Kaiwa Y., Namiki K., Matsumoto H. (2003). Laparoscopic relief of reduction en masse of incarcerated inguinal hernia. *Surgical Endoscopy*.

[7] Abramson J. H., Gofin J., Hopp C., Makler A., Epstein L. M. (1978). The epidemiology of inguinal hernia. A survey in western Jerusalem. *Journal of Epidemiology and Community Health*.

[8] Neuhauser D. (1977). *Elective Inguinal Herniorrhaphy Versus Truss in the Elderly*.

[9] Aytaç B., Çakar K. S., Karamercan A. (2004). Comparison of Shouldice and Lichtenstein repair for treatment of primary inguinal hernia. *Acta Chirurgica Belgica*.

[10] Bhattacharjee P. K. (2006). Surgical options in inguinal hernia: which is the best. *Indian Journal of Surgery*.

[11] Amid P. K. (2004). Lichtenstein tension-free hernioplasty: its inception, evolution, and principles. *Hernia*.

[12] Kurzer M., Belsham P. A., Kark A. E. (2003). The Lichtenstein repair for groin hernias. *The Surgical Clinics of North America*.

[13] Sakorafas G. H., Halikias I., Nissotakis C. (2001). Open tension free repair of inguinal hernias; the Lichtenstein technique. *BMC Surgery*.

[14] Lichtenstein I. L., Shulman A. G., Amid P. K., Montllor M. M. (1989). The tension-free hernioplasty. *The American Journal of Surgery*.

[15] Muldoon R. L., Marchant K., Johnson D. D., Yoder G. G., Read R. C., Hauer-Jensen M. (2004). Lichtenstein vs anterior preperitoneal prosthetic mesh placement in open inguinal hernia repair: a prospective, randomized trial. *Hernia*.

[16] Khoshnevis J. Zh., Falah A. (2013). Comparison of results and complications of Bassini, Liechtenstein and Bassini with mesh techniques in inguinal hernia repair at Shohadaye Tajrish Hospital, Tehran. *Iranian Journal of Surgery*.

[17] Moghaddam J. A., Mehrvarz S., Mohebbi H. A., Panahie F. (2011). Comparison of read-rives and “lichtenstein” repair for treatment of unilateral inguinal hernia. *Koomesh*.

[18] Khorshidi H. R., Azimian M. H., Fazlian M. M. (2007). The Bupivacaine effects on opioids usage and admission duration in patients underwent inguinal hernia repair. *Armaghane Danesh*.

[19] Mohebi H. A., Mehrvarz S. H., Mousavi Naeini S. M., Hosseini Houshyar S. H. (2010). Comparison of quality of life and complications after different surgical methods of unilateral inguinal hernia. *Kowsar Medical Journal*.

